# Real-Time Identification of Smoldering and Flaming Combustion Phases in Forest Using a Wireless Sensor Network-Based Multi-Sensor System and Artificial Neural Network

**DOI:** 10.3390/s16081228

**Published:** 2016-08-04

**Authors:** Xiaofei Yan, Hong Cheng, Yandong Zhao, Wenhua Yu, Huan Huang, Xiaoliang Zheng

**Affiliations:** 1 School of Technology, Beijing Forestry University, Beijing 100083, China; yanxiaofei_21@163.com (X.Y.); yuwenhua56@sina.com (W.Y.); huan2020@foxmail.com (H.H.); valin912@gmail.com (X.Z.); 2 College of Information Science and Technology, Agricultural University of Hebei, Baoding 071001, China; chenghong@cau.edu.cn

**Keywords:** identification, smoldering combustion, flaming combustion, artificial neural network, ZigBee

## Abstract

Diverse sensing techniques have been developed and combined with machine learning method for forest fire detection, but none of them referred to identifying smoldering and flaming combustion phases. This study attempts to real-time identify different combustion phases using a developed wireless sensor network (WSN)-based multi-sensor system and artificial neural network (ANN). Sensors (CO, CO_2_, smoke, air temperature and relative humidity) were integrated into one node of WSN. An experiment was conducted using burning materials from residual of forest to test responses of each node under no, smoldering-dominated and flaming-dominated combustion conditions. The results showed that the five sensors have reasonable responses to artificial forest fire. To reduce cost of the nodes, smoke, CO_2_ and temperature sensors were chiefly selected through correlation analysis. For achieving higher identification rate, an ANN model was built and trained with inputs of four sensor groups: smoke; smoke and CO_2_; smoke and temperature; smoke, CO_2_ and temperature. The model test results showed that multi-sensor input yielded higher predicting accuracy (≥82.5%) than single-sensor input (50.9%–92.5%). Based on these, it is possible to reduce the cost with a relatively high fire identification rate and potential application of the system can be tested in future under real forest condition.

## 1. Introduction

Forest fire has occurred in different regions of the world [1], which entails greenhouse gas emission, pollution and water contamination as well as loss of nutrients and ground microorganisms [2]. Thus, early detection of forest fire is of importance to decrease the loss of natural resource and economical cost. On the other hand, high accurate identification of combustion phases can be of benefit to users for predicting spread direction and speed of forest fire.

Human observation is a traditional method to detect forest fire, but perilous conditions when fire occurred make people flinching. Thus, various novel sensing technologies and tools have been developed instead of human observation of forest fire, such as machine vision-based charge-coupled device (CCD) cameras and infrared (IR) detectors, lidar detection technique, satellite-based remote sensing, wireless sensor networks, etc.

Machine vision method can monitor variation of fire or smoke in forest and report it to a control center [3,4]. The accuracy of machine vision-based system is highly disturbed by terrain, weather conditions (clouds and rain) and smoke from industrial production or social activities. Satellite-based remote sensing is an alternative technique for detecting fire in forest and post-fire recovery management [5,6,7,8]. Remote sensing images are normally scanned by satellites at an interval of 1 or 2 days. Recently, an innovative Himawari-8 geostationary satellite operated by the Japan Meteorological Agency can detect fire hotspots at a 10-min resolution and return data over an entire hemisphere [9,10], which is acceptable for real-time detection. However, one pixel of this remote sensing-based image represents a wide area of around 0.1 hectare with localization error of about 1 km, failing to detect fire or smoke at the beginning of fire occurrence.

Wireless sensor network (WSN) is another alternative technique that becomes more and more popular for real-time monitoring of forest fire [11,12,13,14,15,16,17,18,19,20]. WSN usually consists of some solar-powered nodes integrated with low-cost sensors and modules that can collect important environmental factors, such as air temperature, humidity, air pressure, wind direction and speed, smoke, gas concentration (CO_2_, CO) etc. Then these data are stored and processed, and communicated with a control center through some sink nodes of wireless network. Consequently, if forest fire occurred, the control center can immediately detect the fire. However, when new network architecture is used for deployment of sensor nodes [13,17,19,20], limitations of power supply, storage volume and communication distance must be considered and the network needs to be carefully designed.

Sensors not only can monitor dynamic and static parameters but also allows real-time determining direction and possible evolution of smoke spread and flame front in forest, which are very useful for detecting fires and eradicating them. To detect forest fires with high accuracy and efficiency, some previous studies focused on monitoring chief parameters close related to forest fires [21,22,23,24], such as air temperature, smoke concentration/density etc., and then combined these sensor-based data with machine learning methods. For instance, the literature [21] detected smoke from smoldering combustion in forest combining remote sensing imagery data with artificial neural network and threshold approaches. The literature [23] proposed combining video data from CCD camera with support vector machines and wavelets to detect fire smoke and apply it for smoldering combustion detection in forest and other environment. Similarly, the literature [24] used CCD camera and support vector machines to detect flaming combustion in diverse environment including forest. Although diverse sensing techniques have been developed and combined with machine learning methods for forest fire detection, none of them referred to identifying smoldering and flaming combustion phases in forest. More importantly, the relative contribution of flaming and smoldering combustion is strongly affecting combustion efficiency (CE), with a higher CE indicating more flaming [25,26]. Flaming combustion converts the C, H, N, and S in fuel into highly oxidized gases such as CO_2_, H_2_O, NOx, and SO_2_, respectively, and produces most of the black (or elemental) carbon particles. Smoldering combustion produces most of the CO, CH_4_ and primary organic aerosol. Smoldering and flaming combustion phases frequently occur simultaneously during a fire and are difficult to distinct [27]. For this reason, we tried to identify dominant combustion phase in real-time with a combined use of a developed ZigBee-WSN-based multi-sensor system and artificial neural network (ANN). Outdoor experiment is conducted for testing the responses of the developed multi-sensor system under no, smoldering-dominated and flaming-dominated combustion conditions, respectively. To identify the combustion phases with trade-off between accuracy and cost of the multi-sensor system, artificial neural network (ANN) is applied and tested with different number of sensor inputs.

## 2. Materials and Methods

### 2.1. General Description of the Developed ZigBee-WSN-Based Multi-Sensor System and Its Potential Deployment Strategy in Forest Environment

**Figure 1:**
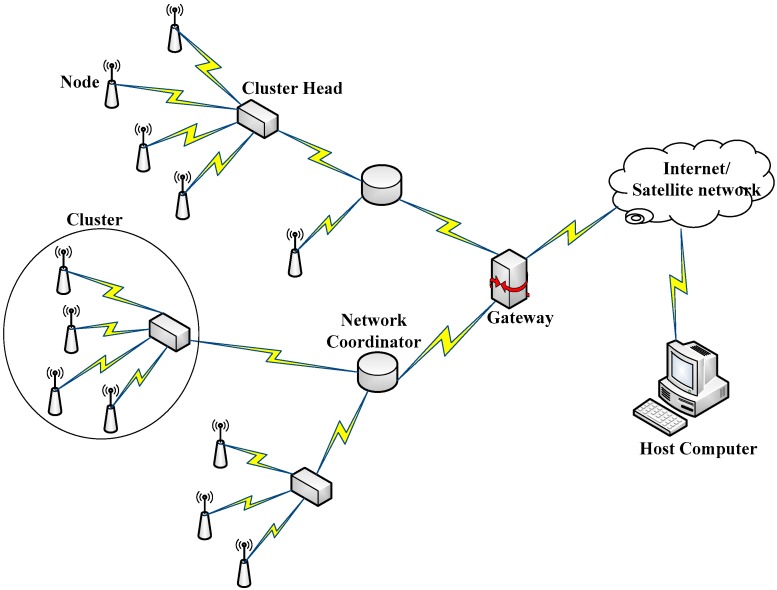
Structure of the developed ZigBee-WSN-based system for forest management and protection, consisting of nodes, cluster heads, coordinators, routers and remote decision server.

Figure 1 schematically shows structure of the developed ZigBee-WSN-based system for forest management and protection, consisting of multi-sensor nodes, cluster heads, coordinators, routers and remote decision server. The cluster-tree network topology structure was applied to reduce the loss of energy and data package. ZigBee technique is a global standard based the IEEE 802.15.4 applicable to low-rate wireless Personal Area Networks. ZigBee is the wireless network standard targeted at low power sensor applications and has dual frequencies 868/915 MHz and 2.4 GHz. The data rates for 868 MHz, 915 MHz and 2.4 GHz frequencies are 20 kbps, 40 kbps and 250 kbps respectively [28,29]. The technical advantage of ZigBee is to offer a system with long battery life, low-cost, small size, high reliability and automatic or semi-automatic installation. Therefore, it is an optimal choice for forest fire monitoring and control. In this study, 2.4 GHz with the data rate of 250 kbps was used [30].

**Figure 2:**
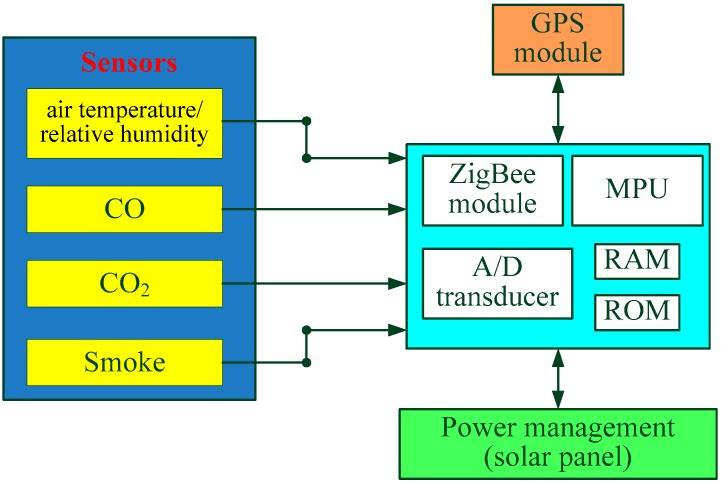
The frame of the multi-sensor node, consisting of a ZigBee RF chip (including an RF wireless transceiver, a micro processing unit, A/D transducer, RAM and ROM), a solar panel, a GPS unit and a series of sensors (CO, CO_2_, smoke, temperature and relative humidity).

The sensor node, as shown in Figure 2, consists of a ZigBee RF chip (CC2430, Chipcon Company [31], price: 12.00 USD) (including an RF wireless transceiver, a micro processing unit (MPU), A/D transducer, RAM and ROM), a solar panel (multi-crystalline silicon, 12 V 7 W, size: 35 cm × 8.8 cm, price: 4.90 USD), a GPS unit (LeadTek GPS 9546, price: 22.00 USD), a CO concentration sensor (EC805-CO, NeMOTO, Japan, accuracy: ±20 ppm, scale: 0–1000 ppm, price: 80.00 USD), a CO_2_ concentration sensor (S-100, ELT, South Korea, accuracy: ±30 ppm, scale: 0–5000 ppm, price: 35.80 USD), a smoke concentration sensor (MS5100, OGAM, South Korea, scale: 0–2000 ppm price: 13.00 USD) and an air temperature/relative humidity sensor (SHT11, SENSIRON, Swiss, price: 4.40 USD). The system based on CC2430 chip is not necessary to transfer data from radio to microcontroller because the microcontroller can access radio registers directly. Positions of the nodes that can be repositioned by GPS could be changed at any time, facilitating its application in forest. The MS5100 can detect aerosol particulates in the air with a small size but high sensitivity, good stability and fast response time to smoke. The accuracy of temperature is up to ±2.5 °C between −40 to 120 °C. The accuracy of relative humidity (RH) sensor is ±3% between 20% and 80% RH, up to ±5% outside of that range. The total cost of each multi-sensor node is about 175 USD.

As well known, the deployment of sensor nodes is an important factor that affects the performance of a WSN system in fire detection [19]. Regular or random deployments are normally two general approaches. Among these studies, the square layout of regular deployment strategy proposed by reference [17] is more suitable to the system applied in this study. The reference [19] considered a denser deployment strategy with a short distance, and also addressed that some nodes had to be deployed to distant locations from others due to geographic problem. Consequently, this would result in a significant cost rising even it can guarantee rapid fire detection in large forest. Moreover, the distance between neighboring sensor nodes will also influence the communication efficiency and power supply, thus a compromise deployment strategy needs to be investigated in a future research under real forest condition.

### 2.2. Artificial Neural Network (ANN)

ANN can be defined as a mathematical model designed to reproduce the way in which the brain performs a particular task. It is considered to be a good classifier due to its inherent features such as adaptive learning, robustness, self-organization, and generalization capability. ANN models require several architectural and training parameters to be selected prior to analysis. The aim of using ANN model in this study is to identify smoldering and flaming combustion phases with high accuracy according to the sensor-measured data. In ANN model, dimension of the nodes at input layer is associated with the number of sensor input, representing the normalized features extracted from the sensor output, which will be tested in Section 3.2. The dimension of nodes in output layer is one including three target outputs: “1” corresponding to no occurrence of fire event; “2” to an occurrence of smoldering event and “3” to an occurrence of flaming event. The number of nodes per hidden layer is generally not known a priori for a specific data set and must be empirically determined through an examination of different parameter settings [32]. In this study, there is one hidden layer and the number of nodes in hidden layer is five for the three conditions.

The ANN model was trained iteratively to minimize the performance function of mean square error (MSE) between the network outputs and the corresponding target values. At each iteration, gradient of performance function (MSE) was used to adjust the network weights and biases. Training process would stop if any of these conditions were met. The initial weights and biases of this network were generated automatically by program. The training and test of the ANN model were performed using the Neural Network Toolbox™ in Matlab 7.0, which is considered as an excellent environment for the processing of large datasets and numerical modeling. The true identification rate of the ANN model for each combustion phase is the ratio between number of identified combustion phase and total number of real combustion phase.

### 2.3. Experimental Procedure and Data Processing

A woody biomass briquette developed by reference [33] was used for producing artificial forest fire. This biomass fuel was made of diverse forest derivations such as woody residual after cutting or processing, shoot, plant stem, etc. with a burning rate of 0.5 kg·min^−1^. The burning production includes CO_2_, CO, H_2_O, SO_2_ and ashes. The burning heat value is around 16,747 kJ·kg^−1^ [33].

The experimental site is located in a demo forest farm of Beijing Forestry University. The biomass briquette was burning in three iron barrels (Diameter: 40 cm; Height: 60 cm). The multi-sensor nodes were tested about 1 meter away from the burning barrels and put around 0.5 m higher than the open edge of the barrels. It needs to be emphasized that, regarding any multi-sensor node, its sensitivity (or response time) to detect the fire signal is related to the distance from fire ignition location [19]. The reference [19] reported that the distance of one node to its closest node should be less than 20 m to recognize the fire threat as early as possible. However, the optimistic distance for enhancing the performance of each multi-sensor node also relies on fuel type of the forest, ignition level, slope of the location and the power of wind in real forest environment. The data were acquired simultaneously at an interval of 1 min. The outdoor tests were conducted to investigate the performance of the multi-sensor nodes under no, smoldering and flaming combustion conditions, respectively. In blind testing, the 1160 data set was randomly divided into two groups containing 316 data for training the ANN model and 844 data as test samples. The detailed parameters for ANN training are given in Table 1.

In order to ensure convergence of the ANN model, the input values (*D*_in_) were normalized as *D*_n_ (0 < *D*_n_ < 1) that are computed using Equation (1) as follow:

(1) $$D_{n}=\frac{D_{in}-D_{\min}}{D_{\max}-D_{\min}}$$

where *D*_max_ is the maximum measurement of sensor; *D*_min_ is the minimum measurement of sensor. The normalized data then were randomized and organized in as matrix input for training. The wireless multiple sensors nodes can send a fire alarm through the access points to a central server if the multiple sensors combined with ANN model identifies an occurrence of fire event.

## 3. Results and Discussion

### 3.1. Responses of the Multi-Sensor System

**Figure 3:**
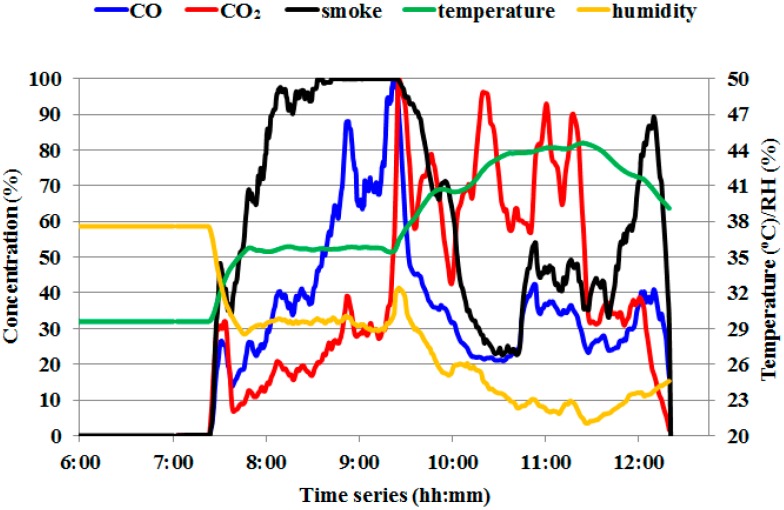
A representative of the responses of the five sensors (normalized concentrations for CO, CO_2_ and smoke sensors) under three different (no combustion/extinguished, smoldering-dominated combustion and flaming-dominated combustion) conditions.

Figure 3 showed a representative of responses of CO, CO_2_ and smoke sensors (normalized concentration) under three different (no, smoldering-dominated and flaming-dominated combustion) conditions. It is notable that (i) the concentrations of CO, CO_2_ and smoke were approximately equal to zero under no combustion condition; (ii) when the fuel was ignited, the condition changed to smoldering-dominated combustion (7:25 a.m.), with the smoke concentration increasing to a value of around 100% (maximum value of the smoke sensor), and meanwhile both CO and CO_2_ concentrations gradually increased in-phase with the concentration of CO higher than that of CO_2_; (iii) However, under the flaming-dominated combustion condition (9:25 a.m.), the CO_2_ concentration increased abruptly to c.a. 80%, accompanied with the simultaneous decrease of smoke and CO concentrations to c.a. 80% and c.a. 30%, respectively, inferring that the CO to CO_2_ ratio can be quite important in determining the dominant combustion phase; (iv) as the fuel was burning out, the transition from flaming-dominated to smoldering-dominated combustion was present, which is similar to the transition in (ii) and finally the combustion was extinguished.

Besides, Figure 3 also showed the same representative of responses of air temperature and humidity sensors under the three different combustion conditions. It is clear to see that (i) the heat production of burning increased the air temperature and decreased the air humidity; (ii) the curve of temperature has a negative relation to that of humidity; (iii) due to larger heat release of flaming-dominated combustion phase (complete combustion), the air temperature was higher than that under smoldering-dominated combustion condition. Because of the effect of daily variations on the amplitudes of temperature and humidity at burning time, the input data of temperature and humidity for ANN were processed by subtracting the values of daily variations.

### 3.2. Performance of ANN Model Associated with Different Input Parameters

Table 2 showed the correlation analysis between the five sensors outputs, indicating that a high positive correlation has been found between the smoke concentration and CO concentration, whereas an extremely high negative correlation between temperature and humidity as expected. Moreover, temperature has correlated with CO_2_ to some extent. Even if the CO to CO_2_ ratio is important in determining the dominant combustion phase, the high positive correlation between CO and smoke leads us to prefer smoke sensor (13.00 USD) rather CO sensor (80.00 USD) as a chief parameter. Due to the relatively low correlation among smoke, CO_2_ and temperature, three chief sensors, smoke, CO_2_ and air temperature, were selected as the inputs for the ANN model.

**Figure 4:**
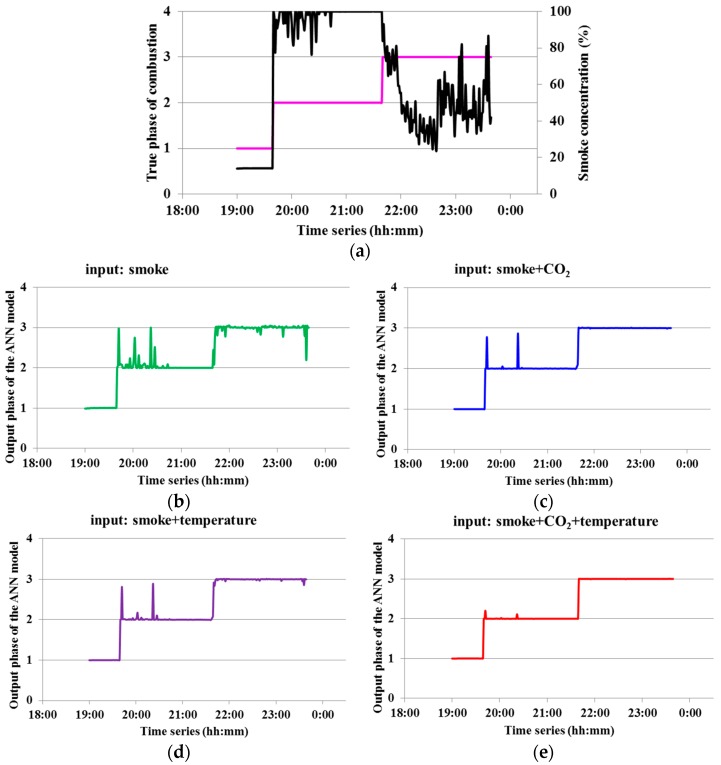
A representative of true combustion phases (**a**) and outputs of combustion phases from the ANN model using single (**b**); two (**c**,**d**) and three (**e**) inputs.

**Figure 5:**
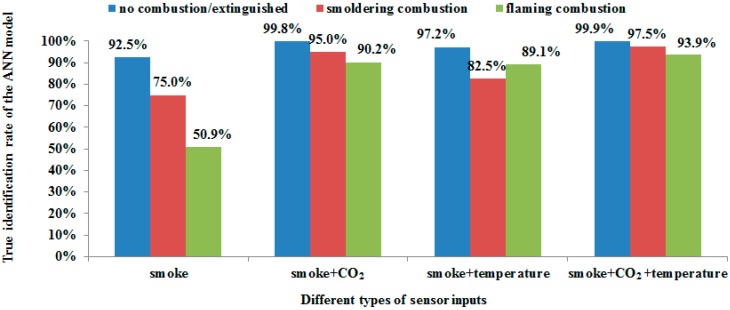
The test results of true identification rate of the ANN model for no combustion/extinguished, smoldering-dominated or flaming-dominated combustion phases under three different input conditions.

To test the ANN model with different sensor inputs, the input layer was modeled using single parameter (smoke), two parameters (smoke and CO_2_; smoke and temperature) and three parameters (smoke, CO_2_ and temperature), respectively. Figure 4 showed a representative of outputs of combustion phases from ANN model using single (Figure 4b), two (Figure 4c,d) and three (Figure 4e) inputs. Figure 4a represented true combustion phase. Comparing Figure 4a with Figure 4b–e, it can be observed that as the input number increased, the classification performance of the ANN model yielded increasing prediction accuracy. Each classification was easily distinguished with the three-sensor inputs. Figure 5 showed the test results of true identification rate of the ANN model for no/extinguished, smoldering or flaming combustion phase under three different input conditions. It can be seen that the multiple sensor inputs can yield higher accuracy (≥82.5%) than the single sensor input (50.9%–92.5%), especially under smoldering-dominated and flaming-dominated combustion conditions. These results also demonstrated the sufficient separation and accuracy in the ANN model.

### 3.3. Potential False Identification Analysis

From Figure 3, it was also revealed the potential false identification for smoldering-dominated and flaming-dominated combustion, which would occur at the relatively long transition time from no to smoldering combustion (transition-1, the red dashed square under smoldering combustion condition) or from smoldering to flaming combustion (transition-2, the blue dashed square under flaming combustion condition). This is confirmed from Figure 4a that most false identification occurred around the transition-1 and transition-2. To minimize this noise, one way could be to enlarge the interval of sampling time, which would result in a limitation of timeliness for real-time monitoring forest fire. Another way could be to include more relevant parameters as input for ANN model, as shown in Figure 4. In general, the multiple inputs of the ANN model are superior to the only one input for identify no, smoldering and flaming combustion phases.

### 3.4. Possibility of Cost Reduction for the Multi-Sensor Node

When considering the use of multi-sensor system in real forest environment, the cost of each node has to be reduced. According to the above results, if only smoke sensor was incorporated into the node for fire detection, the cost would be reduced significantly to about 55.00 USD but the true identification rate would decrease as well (92.5%, 75.0% and 50.9% for no/extinguished, smoldering-dominated and flaming-dominated combustion phases). If smoke, CO_2_ and temperature sensors were used, the cost would be reduced to about 95.00 USD with higher accuracy of fire identification (99.9%, 97.5% and 93.9% for no/extinguished, smoldering-dominated and flaming-dominated combustion phases). As the reference [34] pointed out, wireless sensor node should be relatively cheap with a unit price of less than 100.00 USD for developing countries. Thus, the cost of the node with smoke, CO_2_ and temperature sensors is reasonable and affordable in China. In comparison, if two (group-1: smoke and CO_2_ sensors; group-2: smoke and temperature sensors) sensors were selected, the true identification rate would be higher (99.8%, 95.0% and 90.2% for group-1; 97.2%, 82.5% and 89.1% for group-2) than that using smoke sensor, but lower than that using three chief sensors. In this case, the cost could be reduced to about 90.00 USD for group-1 and 60.00 USD for group-2. In general, it is possible to reduce the cost with a relatively high accuracy according to real forest condition.

## 4. Conclusions

In this paper, we successfully identified different combustion conditions in real time with a combined use of the developed ZigBee-WSN-based multi-sensor system and the ANN prediction model. Three sensors (smoke, CO_2_ and temperature) were chiefly selected through correlation analysis between the outputs of the five sensors (smoke, CO, CO_2_, temperature and relative humidity). The test results of the ANN model showed that the multiple sensor input can yield higher accuracy (≥82.5%) than the single sensor input (50.9%–92.5%). The reason of false identification in ANN model was due to the relatively long transition time from no to smoldering combustion or from smoldering to flaming combustion. Based on these preliminary results, it is possible to reduce the cost with a relatively high accuracy of fire recognition in terms of different forest environments, and the potential application of the developed system combined with the ANN model under real forest condition could be tested in future for fire detection and risk assessment.

## Figures and Tables

**Table 1:** Parameters for training the ANN model.

Training Parameter	Value
Sample	1160
Number of samples for training: 316	
Number of samples for testing: 844	
Input	1 or 2 or 3
Hidden neurons	5
Output neurons	1
Performance	MSE
Goal	0.00001
Learning rate	0.01
Momentum constant	0.9

**Table 2:** Correlation analysis between outputs of the five sensors.

Parameter	CO	CO_2_	Smoke	Temperature	Humidity
CO	1				
CO_2_	0.2808	1			
smoke	0.8008	0.0573	1		
temperature	−0.0872	0.6200	−0.2084	1	
humidity	0.1783	−0.4008	0.2539	−0.9392	1
